# Effects of N-acetylcysteine on spinal cord oxidative stress biomarkers in rats with neuropathic pain

**DOI:** 10.1590/1414-431X20176533

**Published:** 2017-10-19

**Authors:** A. Horst, J.A. de Souza, M.C.Q. Santos, A.P.K. Riffel, C. Kolberg, W.A. Partata

**Affiliations:** 1 Laboratório de Neurobiologia Comparada, Departamento de Fisiologia, Instituto de Ciências Básicas da Saúde, Universidade Federal do Rio Grande do Sul, Porto Alegre, RS, Brasil; 2 Univates, Lajeado, RS, Brasil

**Keywords:** Lipid hydroperoxides, Total antioxidant capacity, Ascorbic acid, Chronic constriction injury, Antinociception

## Abstract

N-acetylcysteine (NAC) inhibits nociceptive transmission. This effect has been associated partly with its antioxidant properties. However, the effect of NAC on the levels of lipid hydroperoxides (a pro-oxidant marker), content of ascorbic acid (a key antioxidant molecule of nervous tissue) and total antioxidant capacity (TAC) is unknown. Thus, our study assessed these parameters in the lumbosacral spinal cord of rats with chronic constriction injury (CCI) of the sciatic nerve, one of the most commonly employed animal models of neuropathic pain. Thirty-six male Wistar rats weighing 200–300 g were equally divided into the following groups: Naive (rats did not undergo surgical manipulation); Sham (rats in which all surgical procedures involved in CCI were used except the ligature), and CCI (rats in which four ligatures were tied loosely around the right common sciatic nerve). All rats received intraperitoneal injections of NAC (150 mg·kg^−1^·day^−1^) or saline for 1, 3, or 7 days. Rats were killed 1, 3, and 7 days after surgery. NAC treatment prevented the CCI-induced increase in lipid hydroperoxide levels only at day 1, although the amount was higher than that found in naive rats. NAC treatment also prevented the CCI-induced increase in ascorbic acid content, which occurred at days 1, 3, and 7. No significant change was found in TAC with NAC treatment. The changes observed here may be related to the antinociceptive effect of NAC because modulation of oxidative-stress parameters seemed to help normalize the spinal cord oxidative status altered by pain.

## Introduction

Neuropathic pain, which is caused by a lesion or disease of the somatosensory system, has high clinical incidence (7–10% of the general population) and seriously affects the quality of life of patients (1). The pathophysiological mechanisms of neuropathic pain are not fully understood (1–3), and the lack of effective analgesics has impelled a continuing search for novel molecules that have beneficial effects in the management of neuropathic pain.

N-acetylcysteine (NAC) is a well-tolerated and safe medication that has been used for several decades as a mucolytic agent and for the treatment of numerous disorders such as acetaminophen intoxication (4,5). It has been demonstrated that NAC has neuroprotective roles (6) and inhibits nociceptive transmission in humans and mice (7). This molecule also induces antinociception in rats with chronic constriction injury (CCI) of the sciatic nerve (8–10). Rat CCI is one of the most commonly employed animal models of neuropathic pain; CCI simulates the symptoms of chronic nerve compression, which correspond to causalgia or complex regional pain syndrome in human patients (11).

The mechanisms responsible for the beneficial effects of NAC have been associated partly with its antioxidant properties. NAC is a cysteine pro-drug and glutathione (GSH) precursor (4,5). GSH is a protective agent and detoxifies reactive oxygen species (ROS) both enzymatically and non-enzymatically (12). NAC has also a direct role in scavenging ROS in neurons (6), which seems to help normalize the oxidative status altered by neuropathic pain, because ROS such as superoxide radicals, hydrogen peroxide (H_2_O_2_) and nitric oxide play an important role in the pathogenesis of neuropathic pain (8,9,10,13). In rats with CCI, NAC treatment reduced nitric oxide metabolites and increased the activity of antioxidant enzymes such as glutathione-S-transferase and glutathione peroxidase in the spinal cord (9).

The main action site for ROS in neuropathic pain is the spinal cord (14), which is part of the central nervous system (CNS). The CNS has large amounts of polyunsaturated fatty acids, and damage to lipids, i.e. lipid peroxidation, caused by ROS, is a very common event, especially when there is oxidative stress. Oxidative stress may be defined as an excessive amount of ROS, which is the net result of an imbalance between production and destruction of these species (15). A complex antioxidant defense system exists to reduce the damage from ROS. One important antioxidant molecule of the CNS is ascorbic acid, which is involved in the first line of antioxidant defense, protecting lipid membranes and proteins from oxidative damage. When ascorbic acid carries out its antioxidant activity, it is oxidized; the reduction of the oxidized form is an enzymatic reaction, which may be glutathione-dependent (16).

Since neuropathic pain increases ROS (8,9,10,13) and NAC is a precursor of GSH (4,5), we postulated that NAC treatment would change the levels of lipid hydroperoxides and ascorbic acid in the lumbosacral spinal cord from CCI rats, the region where most afferent fibers of the sciatic nerve enter. Thus, our study assessed the effect of intraperitoneal administration of NAC (150 mg·kg^−1^·day^−1^), given for 1, 3, and 7, days, on the lipid hydroperoxide and ascorbic acid levels in the lumbosacral spinal cord of rats with CCI. Since oxidative stress has also been determined by the measurements of a decrease in total antioxidant capacity (TAC) (13), we also assessed this parameter in the spinal cord of the rats with CCI. This measurement is important because NAC has an antioxidant action as discussed above.

The dose of NAC (150 mg·kg^−1^·day^−1^) was chosen because it showed antinociceptive effect in rats with CCI (9,10). The time points of the study (1, 3, and 7 days) were based on our previous study with NAC treatment (10).

## Material and Methods

### Animals

All animal procedures were approved by the Animal Ethics Committee of the Universidade Federal do Rio Grande do Sul (#23407). Adult male Wistar rats weighing 200–300 g were divided into three experimental groups (Naive, Sham, and CCI), and each one was further divided into two subgroups (n=6 each), which received NAC (Fluimucil®, Zambon Laboratórios Farmacêuticos Ltda., Brazil) at a dose of 150 mg·kg^−1^·day^−1^ or 0.9% saline solution intraperitoneally for 1, 3, or 7 days. Administration started on the day of surgery (beginning 4 h after recovery from anesthesia) and was performed daily at 5:00 pm by the same researcher (9,10). Rats were not anesthetized for the injections.

### Induction of chronic constriction injury

CCI was performed based on the procedure described by Bennett and Xie (17), with slight modifications according to Horst et al. (9,10). After anesthesia (90 mg/kg ketamine and 10 mg/kg xylazine), the right common sciatic nerve was exposed via a mid-thigh incision. Proximal to the sciatic trifurcation, the nerve was freed of adhering tissue for about 7 mm, and four ligatures (4.0 Shalon chromic catgut, Shalon Fios Cirúrgicos LTDA, Brazil) were tied loosely around it, with a 1.0–1.5 mm interval between each ligature. After nerve ligation, the muscle and skin layers were immediately sutured with thread and a topical antibiotic was applied. To expose the sciatic nerve in sham rats, all surgical procedures involved in CCI were used except the ligature.

### Sample preparation

Rats were killed by decapitation and their lumbosacral spinal cord was promptly dissected, homogenized in 1.15% KCl diluted 1:5 (w/v) containing 1 mmol/L phenylmethylsulfonyl fluoride, and centrifuged at 1000 *g* for 20 min at 4°C. The supernatant was used for assays of lipid hydroperoxides and ascorbic-acid levels and TAC.

### Determination of lipid hydroperoxides levels

The lipid hydroperoxides were measured by oxidation of Fe^2+^ by LOOH in an acid medium containing xylenol orange dye, which forms a complex with Fe^3+^, as described by Jiang et al. (18). Results are reported as nmol/mg protein.

### Determination of ascorbic acid levels

Ascorbic acid (AA) content was determined according to the method described by Roe and Kuether (19). The assay mixture contained 0.3 mL homogenate treated with charcoal and filtered, 0.01 mL 10% thiourea and 0.075 mL 2% DNPH and was incubated at 37°C for 3 h. Following this, color was produced by adding 0.375 mL 85% sulfuric acid and the absorbance was read at 540 nm. The standard curve was prepared using different concentrations of AA and the slope was used to report the amount of AA as µmol of AA/mg protein.

### Determination of TAC

The TAC was determined with 2,2-azinobis-(3-ethylbenzothiazoline-6-sulfonic acid radical cation (ABTS*^+^), which in an acid medium is decolorized by antioxidants, according to their concentration and antioxidant capacity (20). Results are reported as mmol·eq trolox^-1^·g tissue^-1^.

### Protein measurement

Protein was measured by the method of Lowry et al. (21), using bovine serum albumin as the standard.

### Statistical analysis

The results were analyzed using three-way ANOVA (factors: lesion, treatment, and time) followed by the Tukey *post hoc* test. Differences were considered statistically significant when P<0.05.

## Results

After CCI, all rats exhibited a decrease in mechanical threshold, which was prevented by NAC treatment, as shown in our previous study (10).

**Figure 1.**
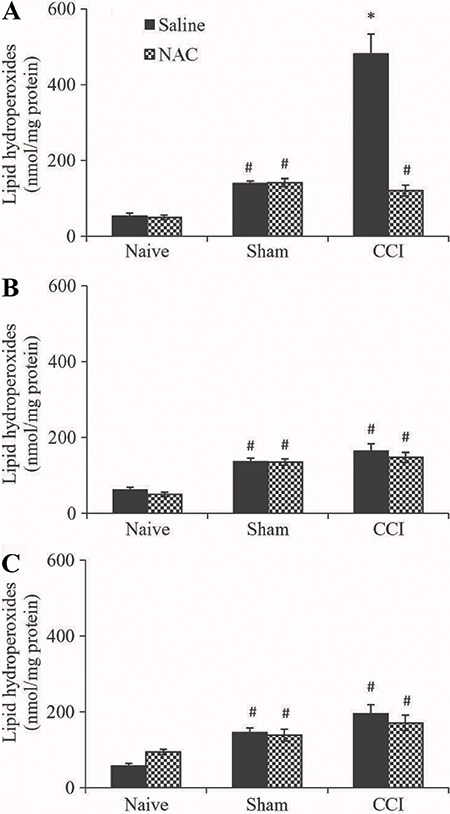
Lipid hydroperoxide levels in the spinal cord of chronic constriction injury (CCI) rats treated with N-acetylcysteine (NAC; 150 mg·kg^−1^·day^−1^) or saline administered intraperitoneally for 1 (*A*), 3 (*B*), and 7 (*C*) days (n=6 animals/group). In Sham rats, all surgical procedures involved in the CCI were used except the ligature. Data are reported as means±SE. *P<0.05, compared to other groups. ^#^P<0.05, compared to naive group (three-way ANOVA followed by Tukey *post hoc* test).

After CCI, lipid hydroperoxide levels increased in the spinal cord at day 1. While the increase was 823% in the spinal cord from saline-treated CCI rats, the increase was only 142% in the NAC-treated CCI rats, compared to naive rats (Figure 1A), showing that the NAC treatment sharply reduced the CCI-induced increase in lipid hydroperoxides, at this time point. At postoperative days 3 and 7, the lipid hydroperoxide levels were still increased (180%) in the spinal cord of CCI rats compared to naive rats, but the levels were similar between saline- and NAC-treated CCI rats (Figure 1B and C). Lipid hydroperoxides also increased in the spinal cord from saline- and NAC-treated sham rats, compared to naive rats (180%), at all time points. The lipid hydroperoxide level showed no significant change in the spinal cord of naive rats.

**Figure 2.**
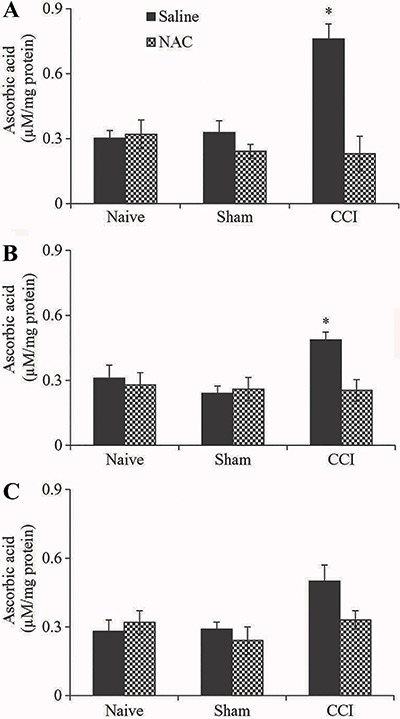
Ascorbic acid levels in the spinal cord of chronic constriction injury (CCI) rats treated with N-acetylcysteine (NAC; 150 mg·kg^−1^·day^−1^) or saline administered intraperitoneally for 1 (*A*), 3 (*B*), and 7 (*C*) days (n=6 animals/group). In Sham rats, all surgical procedures involved in the CCI were used except the ligature. Data are reported as means±SE. *P<0.05, Significant difference compared to other groups. (three-way ANOVA followed by Tukey *post hoc* test).

CCI induced a significant increase in the ascorbic acid content in the spinal cord of rats that received saline. The increase was 137 and 53% at days 1 and 3, respectively, compared to naive rats (Figure 2A and B). No significant change was found in the levels of ascorbic acid in the spinal cord from CCI rats treated with saline for 7 days (Figure 2C). However, the ascorbic acid level increased by 31% in these rats compared to naive rats. No significant change occurred in ascorbic-acid levels in the spinal cord from NAC-treated CCI rats at all time points (Figure 2A–C). In NAC-treated CCI rats, the ascorbic acid content was similar to that found in saline- and NAC-treated naive rats. This similarity suggests that NAC treatment prevented a CCI-induced increase in ascorbic-acid content. No significant change was found in the ascorbic acid levels of naive and sham rats treated with NAC or saline for 1, 3, and 7 days.

**Figure 3.**
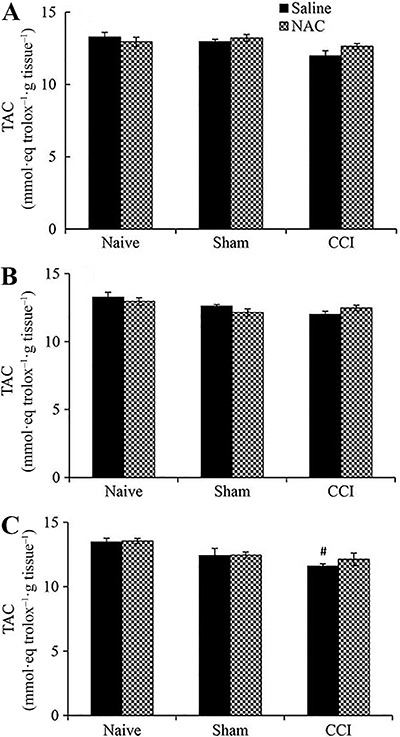
Total antioxidant capacity (TAC) in the spinal cord of chronic constriction injury (CCI) rats treated with N-acetylcysteine (NAC; 150 mg·kg^−1^·day^−1^) or saline administered intraperitoneally for 1 (*A*), 3 (*B*), and 7 (*C*) days (N= 6 animals/group). In Sham rats, all surgical procedures involved in the CCI were used except the ligature. Data are reported as means±SE. ^#^P<0.05, compared to naive group (three-way ANOVA followed by Tukey *post hoc* test).

TAC did not show significant changes in the spinal cord from saline- and NAC-treated CCI rats at days 1 and 3 (Figure 3A and B). At day 7, NAC treatment did not produce a significant change in TAC of the spinal cord from CCI rats, but saline-treated CCI rats showed a significant decrease (11%) in this parameter (Figure 3C). No significant change was found in TAC in the spinal cord from sham and naive rats.

## Discussion

Neuropathic pain is a disease of global burden and its prevalence has been estimated to be in the range of 7–10% (1,3). It is suggested that the incidence of neuropathic pain is likely to increase owing to the ageing global population, increased incidence of diabetes mellitus and improved survival from cancer after chemotherapy (1). Diabetic polyneuropathy and neuropathies associated with chronic low back pain are the most frequent causes of neuropathic pain (3). Numerous therapeutic recommendations for neuropathic pain have been proposed (pregabalin, gabapentin duloxetine and various tricyclic antidepressants), but most of these treatments have moderate efficacy based on the dose needed to treat the patient (1). Thus, it is important to find new compounds that may be used to treat neuropathic pain.

Our results showed that NAC treatment prevented the early CCI-induced increase in lipid hydroperoxide levels of the lumbosacral spinal cord, without changing the levels of ascorbic acid and TAC in this tissue, which were increased and decreased, respectively, after CCI (11). The high spinal cord lipid hydroperoxide levels from saline-treated CCI rats at day 1 might be related to an increase in CCI-induced ROS formation. A recent study showed that superoxide-anion generation (SAG) increased by around 40% in the spinal cord of CCI rats treated with saline for 1, 3, and 7 days (10). In the central nervous system, damage to lipids is a very common event caused by ROS, due to the large amounts of polyunsaturated fatty acids (15). Thus, the increase in SAG may have contributed to the elevation in lipid hydroperoxide levels in the spinal cord after CCI. The lower formation of lipid hydroperoxides in the spinal cord from saline-treated CCI rats at days 3 and 7 may be related to mobilization of antioxidant systems. Catalase, an antioxidant enzyme that catalyzes the breakdown of H_2_O_2_ to H_2_O and O_2_ (20), increases its activity in the spinal cord 3 and 10 days after CCI (22).

NAC treatment, in turn, prevented an accentuated increase in lipid hydroperoxides in the spinal cord from CCI rats at day 1. Lipids are important targets of ROS, as described above. The reduction in lipid hydroperoxide formation suggests that ROS decreased after NAC treatment. NAC is a precursor of GSH, an important intracellular antioxidant (4,5,12), and can directly scavenge ROS in neurons (6). These antioxidant actions of NAC may contribute to decrease ROS in the spinal cord. This reduction could lead to less lipid peroxidation, which may explain the decrease in lipid hydroperoxide levels in the spinal cord from NAC-treated CCI rats after 1 day. Another explanation may be the NAC action on calcium influx. NAC has a protective role in calcium influx through transient receptor potential melastatin-like 2 (TRPM2) channels (6). According to Sözbir and Nazıroğlu (6), dorsal root ganglion neurons exhibit TRPM2 channel-dependent ROS generation and calcium influx in neuropathic pain. Thus, it is necessary to consider that the preventive NAC action on lipid peroxidation may be related to its action on calcium influx through TRPM2 channels. The antioxidant action of NAC for 3 and 7 days of treatment may also help maintain the levels of lipid hydroperoxides similar to those found on day 1.

Interestingly, lipid hydroperoxides were still significantly increased in the spinal cord of NAC-treated CCI rats at days 3 and 7 compared to naive rats, and the levels were similar to those found in saline-treated CCI rats. Excessive ROS formation needs to be corrected only to prevent the accumulation of oxidative damage, and a slight pro-oxidative balance is necessary for optimal cell-signaling processes (23). Thus, it is probable that the elevated lipid hydroperoxide levels in the spinal cord from saline- and NAC-treated CCI rats are related to maintenance of necessary conditions for cell-signaling processes. Lipid peroxidation is essential for phospholipase C activity and the inositol-triphosphate-related calcium signal (14), which are signaling mechanisms activated when different molecules exert their nociceptive (1,2,3) and antinociceptive (24) actions.

The lipid hydroperoxide levels were also elevated in the spinal cord of saline- and NAC-treated sham rats for 1, 3, and 7 days. This increase may be related to surgery-induced ROS formation. These rats were submitted to procedures involving manipulation of deep tissues, such as muscles and adjacent connective tissue, which induce pain (25). Thus, the higher lipid hydroperoxide levels in the spinal cord from sham rats may also be related to actions of the molecules discussed above. The difference found in the spinal cord from sham and CCI rats that received saline for 1 day might be related to neuronal sensitization, a key mechanism in the pathology of neuropathic pain. According to Goecks et al. (22), the differences in oxidative-stress parameters between sham and CCI rats could be related to different degrees of sensitization of the nervous tissue in these groups of rats.

In our study, while ascorbic-acid levels increased in the spinal cord of the saline-treated CCI rats for 1, 3 and 7 days, NAC treatment prevented an increase in this tissue at these time points. This difference may be related to the roles described for ascorbic acid in nervous tissue. In this tissue, ascorbic acid acts as an important antioxidant molecule, a neuromodulator of synaptic activity, and it functions in the metabolic switch of the neurons during brain activity and resting conditions, having its levels increased in response to brain activity (16). Accumulating evidence from diverse animal models of neuropathic pain suggests that neuropathic pain might involve aberrant excitability in the dorsal horn, resulting from multiple functional alterations including increases in the release of glutamate and other neurotransmitters, as well as in ROS formation, loss of function of inhibitory interneurons, and multiple alterations in glial and immune cells of the CNS (1–3,26–28). Since CCI is a model of neuropathic pain (11), these changes probably occurred in the spinal cord of rats with CCI. Thus, it is probable that the increase in ascorbic acid levels may be related to CCI-induced changes in the spinal cord from saline-treated CCI rats. Neurons are highly sensitive to oxidative damage, and mechanisms to maintain antioxidant activity are required during physiological activities such as recycling and release of ascorbic acid by astrocytes (16).

NAC causes the activity of the L-cystine/L-glutamate membrane exchanger, and L-cysteine is required for the synthesis of the GSH (29,30). These authors showed that the activity of the L-cystine/L-glutamate membrane exchanger reduced after CCI. In addition, protracted oxidative stress and diminished antioxidant defenses are associated with GSH oxidation and depletion (12), a phenomenon observed in diabetic neuropathic pain (6) and CCI-induced neuropathic pain (31). Thus, NAC-induced GSH restoration might explain the lack of a significant increase in ascorbic-acid levels in the spinal cord of the CCI rats that received NAC treatment. Although our study did not assess GSH levels, its results stress the need to assess this parameter for better understanding the relation between GSH and changes in lipid hydroperoxides and ascorbic acid.

Since NAC is a precursor of GSH (4,5), it appears important to determine if NAC treatment increases antioxidant defenses in the spinal cord. According to Poljsak et al. (23), the balance between ROS and antioxidants is necessary, because both extremes, oxidative and antioxidative stress, are damaging. TAC showed no significant change in the spinal cord from CCI rats after NAC treatment. This may be related to tight control of intracellular conditions when there is antioxidant supplementation. Thus, the TAC result may be indicating that the NAC treatment did not disrupt the well-integrated antioxidant defense networks.

However, TAC decreased in the spinal cord from saline-treated CCI rats at day 7. It has been demonstrated that TAC represents the enzymatic and non-enzymatic antioxidant compounds in the body such as superoxide dismutase, catalase, glutathione peroxidase and GSH (32,33). Some of these antioxidant parameters are reduced in neuropathic pain (6,22,31), while other, as catalase activity, increase after CCI (22). Nitroxidative species (ROS, reactive nitrogen species and their products) can directly increase the excitability of nociceptive neurons (34). The interrelated changes in pro-oxidant and antioxidant defenses may be related to lack of significant changes in TAC at days 1 and 3 and the significant decrease in this parameter at day 7. However, antioxidant depletion does not necessarily mean that oxidative damage has taken place; it might simply mean that the defense mechanisms have removed ROS and protected the system (15). Since ROS are involved in the pathogenesis of neuropathic pain (6–10,13,19,22,25,27,28,34), it may be suggested that the increase in ROS may be related to a decrease in TAC in the spinal cord of saline-treated CCI rats.

The lack of significant changes in ascorbic acid levels and TAC in the spinal cord of the sham rats may be related to differences in the degree of central sensitization in sham and CCI rats, as suggested above. According to Goecks et al. (22), CCI injury, differently from the sham condition, probably mobilizes the antioxidant system to a greater degree, in order to prevent the establishment of an oxidative-stress situation, given the greater excitation of central sensory neurons.

In conclusion, our study provides evidence that NAC treatment prevented the early CCI-induced increase in lipid hydroperoxide levels of the lumbosacral spinal cord while preventing the rise of ascorbic acid content, and the decrease of TAC at later stages. Since NAC has an antinociceptive effect in rats with CCI as shown in our previous study (10), the changes observed here may be related to this effect of NAC because modulation of oxidative-stress parameters seems to help normalize the spinal cord oxidative status altered by pain.
